# Predicting human odor perception represented by continuous values from mass spectra of essential oils resembling chemical mixtures

**DOI:** 10.1371/journal.pone.0234688

**Published:** 2020-06-19

**Authors:** Tanoy Debnath, Takamichi Nakamoto

**Affiliations:** 1 Department of Information and Communications Engineering, Tokyo Institute of Technology, Yokohama, Kanagawa, Japan; 2 Laboratory for Future Interdisciplinary Research in Science and Technology, Tokyo Institute of Technology, Yokohama, Kanagawa, Japan; Instituto Nacional de Medicina Genomica, MEXICO

## Abstract

There have been recent advances in predicting odor characteristics using molecular structure parameters of chemicals. Although the molecular structure parameters are available for each chemical, they cannot be used for chemical mixtures. This study will elucidate a computational method of predicting human odor perception from the mass spectra of chemical mixtures such as essential oils. Furthermore, a method for obtaining similarity among odor descriptors has been proposed although the dataset contains binary values only. When the database indicates a set of odor descriptors for one sample, only binary data are available and the correlation between the similar descriptors disappears. Thus, the prediction performance degrades for not considering the similarity among the odor descriptors. Since mass spectra dataset is highly dimensional, we use auto-encoder to learn the compressed representation from the mass spectra of essential oils in its bottleneck hidden layer and then accomplishes the hierarchical clustering to create odor descriptor groups with similar odor impressions using a matrix of continuous value-based correlation coefficient as well as natural language processing. This work will help to expatiate the process of overcoming binary value problem and find out the similarity among odor descriptors using machine learning with natural language semantic representation of words. To overcome the problem of disproportionate ratio of positive and negative class for both the continuous value-based correlation coefficient and word similarity based models, we use Synthetic Minority Oversampling Technique (SMOTE). This model allows us to predict human odor perception through computer simulations by forming odor descriptors group. Accordingly, this study demonstrates the feasibility of ensembling machine learning with natural language processing and SMOTE approach for predicting odor descriptor group from mass spectra of essential oils.

## Introduction

Out of five major human senses, olfaction and taste are responsible for chemical perception and recognition. The odor is the result of perceiving a chemical stimulus using the sense of smell. The source of odor is a molecule that exists in the air and these odorants attach to and activate olfactory receptors (ORs) on an ORN (Olfactory Receptor Neuron) [1], which sends the odor signal to the olfactory bulb, and the reaction pattern is created in the brain. The biological understanding of odor perception in the sense of smell has made remarkable progress since the discovery of odor receptors by Buck and Axel in 1991 [2].

Since then, researchers have made great progress in predicting olfactory perception using molecular structure parameters such as molecular weight and functional groups. In the previous studies, molecular structure parameters [3,4], activation information of the olfactory bulb [5] were used as inputs to the neural network model. It is feasible to collect molecular structure parameter for each chemical by dedicated software, but in real life, the odor is represented as a mixture rather than a pure single molecule substance. It is not possible to construct a model that can be used as a mixture for the inputs of the neural network, because the parameters of the molecular structure cannot be used to express the mixture.

In the present study, mass spectra of essential oils have been adopted as a chemical mixture. Since fragment peaks of mass spectra contain useful information on molecular structure, they can be used as predictors for neural network model. In addition, since the mass spectrum can express the mixture, it is possible to predict the odor perception of the chemical mixture such as an essential oils much easier than the molecular structure parameters. In the field of industrial products such as food, cosmetics and consumer products, adjustment of blending ratio of many samples is carried out daily by trial and error by perfumers and flavorists which needs much cost and takes long time. Automation for identifying the odor impression and prediction will be significant in this area.

Additivity is not required to be considered since we have a mass spectrum corresponding to certain essential oil which is the mixture of many chemicals. We do not mix samples in this paper even if each sample is the mixture of chemicals. Since essential oils are a complex mixture of natural, volatile and aromatic compounds derived from plants, an essential oil can be described with different odor descriptors. Thus, our target is to cluster the similar kind of odor descriptors [6] in same odor space and then predict the human odor perception using machine learning where the model will predict the presence or absence of odor descriptor group from the mass spectra of chemical mixtures such as essential oils. Previous article showed the method of predicting odor character from the mass spectrum of the NIST dataset and the chemical list of Sigma-Aldrich catalog [7]. But the dataset used in that study contains only a set of odor descriptors for an odorant, which means binary value data accompanied with a problem for vanishing correlation between similar odor descriptors. Similar descriptors (such as ‘floral’ and ‘rose’) have a tendency to be mutually exclusive and therefore we cannot find similarities between the odor descriptors. That’s why in our current study, we try to solve this problem by converting the original binary dataset into continuous value and creating odor descriptor groups among similar odor descriptors. Correlation- coefficient based clustering based on continuous values of odor descriptors and natural language semantic word similarity methods are shown here to form odor descriptor groups.

When a person perceives an odor, it is generally a mixture. The mass spectrum, one of the structural information of the molecule of an odor, is set of the peaks of the respective component molecules. The relationship between mass spectrum and odor impressions perceived by people is not simple, it can be said that there is a strong nonlinear relationship between them. An auto-encoder [8] is a neural network that can extract feature vectors from the original nonlinear high dimensional space of mass spectra [9] into its bottleneck latent space. Then, these reduced feature vectors of mass spectra are used as an input to neural network to predict the odor descriptor groups. Finally, Synthetic Minority Oversampling Technique (SMOTE) is used to balance the imbalanced ratio of positive and negative samples.

In previous PLOS One article [7], we described the method to calculate the correlation coefficient between odor descriptors only using binary data. Then, this method was compared with natural language processing method Word2vec to predict odor characters of chemicals.

Our current paper is an extended work of our previous article in IEEE ISOEN conference [6], where we proposed the method to obtain scent impression scores with continuous values from the score with binary value. Natural language processing was not used there.

In current paper, the method using continuous value based correlation coefficient proposed in ISOEN [6] is compared with natural language processing method Fast-Text. Although the correlation coefficient based on binary value was also used in the previous PLOS One paper [7], it was different from the current one which is based on continuous value. Thus, we replaced the correlation coefficient with continuous value-based correlation coefficient in our current paper to clarify this point.

Moreover, our two previous works did not describe any procedure to overcome the imbalanced ratio of positive (Minority samples with specific odor descriptor group) and negative samples (majority samples with non-specific odor descriptor group) that influenced the model predictive performance. In this paper, we use synthetic minority oversampling technique (SMOTE) to balance the positive and negative samples for both correlation similarity and word similarity based odor descriptor group prediction.

## Mass spectrum dataset for essential oils

The mass spectrum is one of the structural properties of molecules and is given as a plot of intensity vs the mass-to-charge ratio (m/z). The peaks in the mass spectrum of the mixture are based on the molecules of the components. Tiny peaks in the mass spectrum often contribute to the smell, whereas several large peak does not [10,11].

In this study, we use mass spectrum dataset of essential oils collected by Gas chromatography–mass spectrometry [Agilent technologies, 7890 GC system and 5977 MSD using electron ionization (70 eV)]. As this work is concentrated only on mass spectrum, the column without coating was used in this experiment. We use 96 mass spectra of essential oils which can be divided into seven groups [12], namely ‘Herbal’, ‘Citrus’, ‘Floral’, ‘Exotic’, ‘Resin’, ‘Spicy’, ‘Woody’. Intensities of 50–250 m/z were extracted from the original data and were used for these experiments because the intensities of m/z below 50 is derived from odorless molecules such as oxygen, nitrogen and intensities at high m/z originates from molecules with low volatility. Thus, we obtained a data matrix of 96 essential oil samples with 201 intensities. This data matrix of mass spectra was rescaled in the range of 0 and 1 after dividing by the maximum value of the same mass spectrum.

## Proposed method

**Fig 1:**
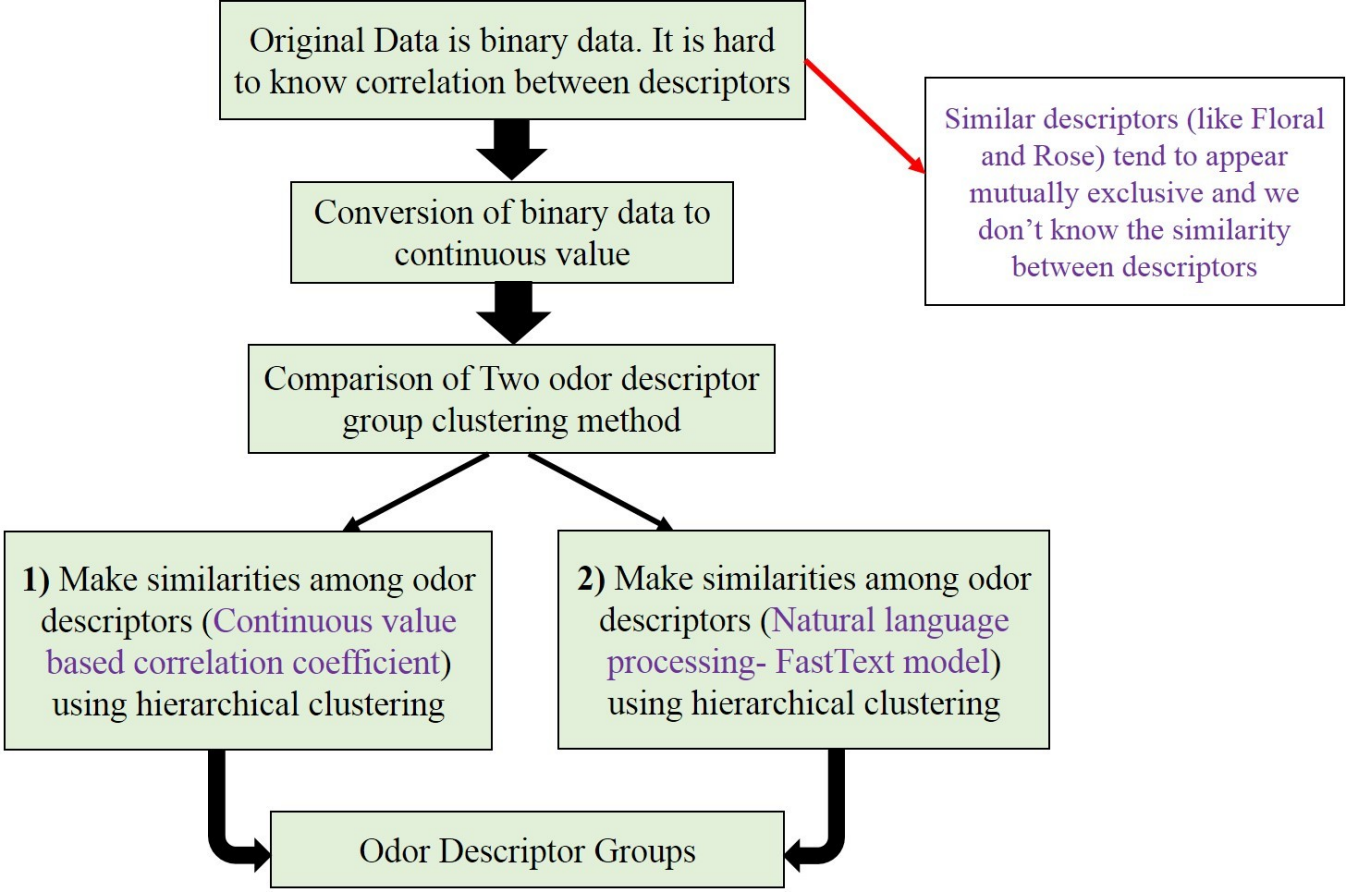
Two proposed approaches to obtain odor descriptor groups. Mass spectra and predictive model are not described in Fig 1.

An auto-encoder followed by a neural network method is used to predict the odor descriptor group of essential oils using mass spectrometry as features. Since our original dataset is binary data, it is hard to know the correlation between descriptors. We simplify this problem in two ways: **1)** One way is to create odor descriptor groups based on a dendrogram built by transforming the binary values of all the descriptors for each essential oil into the continuous values of the 7 types of essential oils and how frequently they appears on each essential oil groups & **2)** another way is to build odor descriptor groups based on the word similarity matrix (Fast-Text model). (Fig 1) depicts our proposed approach to obtain odor descriptor groups.

### Procedure for making seven dimensional vector for odor descriptor

**Fig 2:**
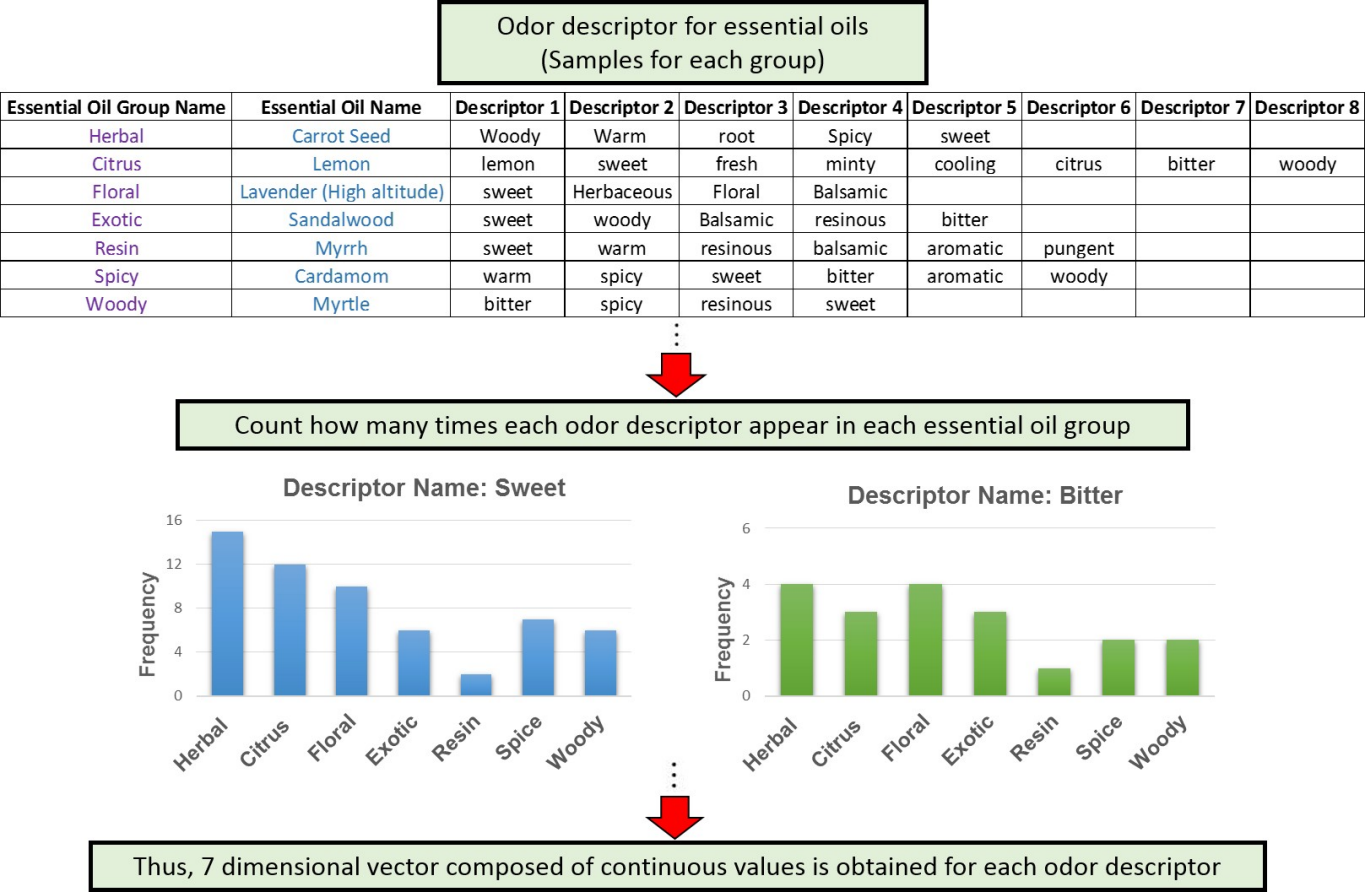
Method to create seven dimensional vector of continuous values for 38 odor descriptors [6].

Seven group of essential oils has been used in this study. Our main goal is to express the dataset with continuous values even though our original dataset contains binary values only. Procedure for making seven dimensional vector for 38 odor descriptors has been delineated in (Fig 2). At first, a table (S1 Table) of odor descriptors of 96 essential oils was prepared by using ‘Flavor database’ and ‘The Directory of Essential Oil’ by Wanda Sellar [12]. Then, we created a table (S2 Table) using all the essential oils assigned to one of the seven essential oil groups. Next, we examined the number of times each odor descriptor appeared in one of the seven essential oil groups. As a result, even though the original data is binary, a seven dimensional vector can be created for each odor descriptor with integrated components of continuous values. Initially, hundreds odor descriptors were found, but some of them appeared only once. Thus, this type of odor descriptors was excluded from the descriptor list. Then, we obtained 38 odor descriptors from 96 essential oils of seven essential oil group.

#### Odor descriptor clustering method

A hierarchical clustering method was applied using a dendrogram to obtain a better predictive model. A dendrogram helps to find the clustering that is most applicable for an application. This study compares two types of odor descriptor clustering methods, 1) a matrix of correlation coefficient based on continuous values and 2) word similarity matrix using natural language processing. In this paper, Fast-Text created by Facebook’s AI research [13] was used for making similarity of odor descriptor group.

#### Odor descriptor clustering using a matrix of correlation coefficient based on continuous values

Several odor descriptors are available for each essential oil, and it is important to consider the similarities among those 38 odor descriptors of seven essential oil groups. In order to understand the impression of the same odor under different names, it is necessary to make hierarchical clustering of similar odor descriptors because it is the hierarchical decomposition of the data based on group similarities. Dendrogram clustering on correlation coefficient matrix was performed on the basis of unweighted average distance method. Several odor descriptor group are possible when creating the cluster of similar odor descriptors using the correlation coefficient matrix. Thus, we considered 5 odor descriptor groups in this study.

#### Odor descriptor clustering using word similarity matrix

We used pre-trained word vectors trained on English Wikipedia using Fast-Text to accurately evaluate the semantic similarity among odor descriptors. 300 dimensional vectors were obtained using the skip gram model described in [14] with the default parameters. Here, the word vector has 300 dimensions and the number of tokens (words) in a vector file varies from language to language. English pre-trained word vector model was downloaded (4.20 GB) and there are 2000000 tokens available. After that, the cosine similarity of 38 odor descriptors was calculated using pre-trained English wiki Fast-Text model. Finally hierarchical clustering was carried out based on cosine similarity matrix.

#### Result of hierarchical clustering analysis

**Fig 3:**
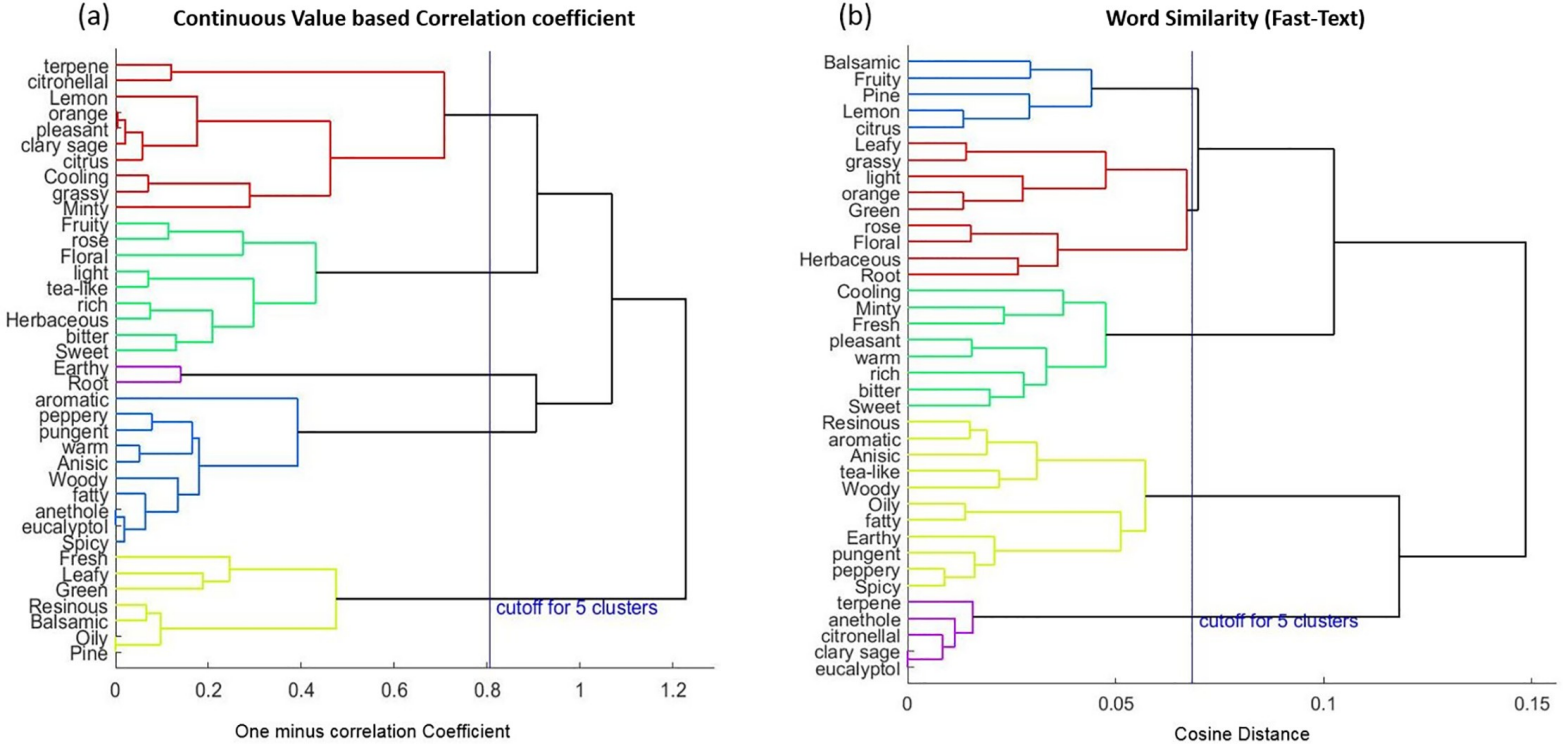
Dendrograms (Unweighted average distance method) based on, a) One minus the sample correlation between two odor descriptors, b) Cosine distance based on Fast-Text modeling. The vertical line in both figures indicates the cut off point for 5 clusters.

Fig 3(A) and 3(B) illustrate a hierarchical structures using the dendrograms obtained from the correlation between two odor descriptors and cosine distance based word similarity matrix. Horizontal axis of the dendrogram represents the dissimilarity between descriptors. Each cluster comprises of several odor descriptors and the number of these clusters are determined at a cutoff point. For example, the number of clusters is 5 as shown in Fig 3(A) and 3(B).

### Predictive model for odor descriptor group with clustering

**Fig 4:**
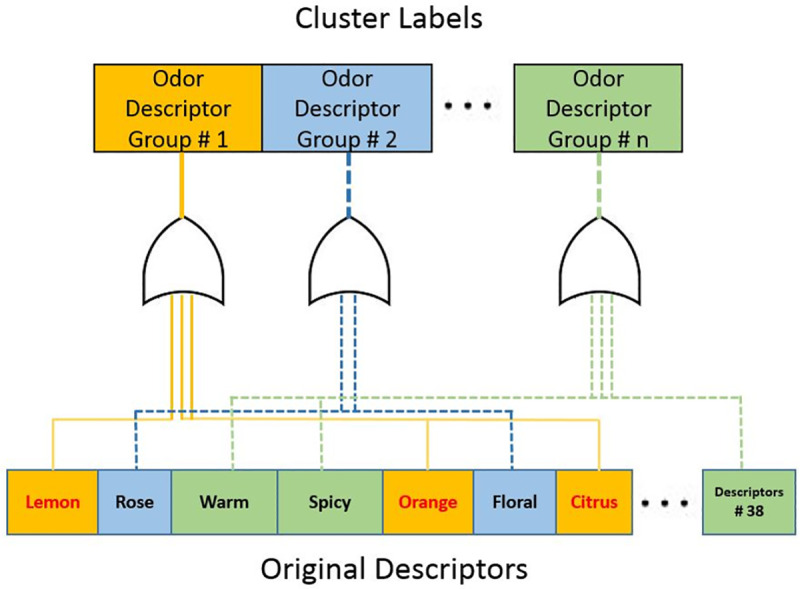
Odor descriptor group labeling procedure for the predictive model. When orange, citrus, lemon included in a particular cluster, an essential oil having at least one of these descriptors in the root descriptors table of essential oils is considered the odor character of that cluster. Here, **# n** indicates the number of odor descriptor group. In this study 4 to 8 odor descriptor group has been used.

The predictive model requires an output vector that indicates the existence of the odor descriptor group. Fig 4 shows the odor descriptor group labeling procedure for the predictive model. For instance, when orange, citrus, lemon are included in a particular cluster, an essential oil having at least one of these descriptors in the root descriptors table of essential oils is considered the odor character of that cluster.

**Fig 5:**
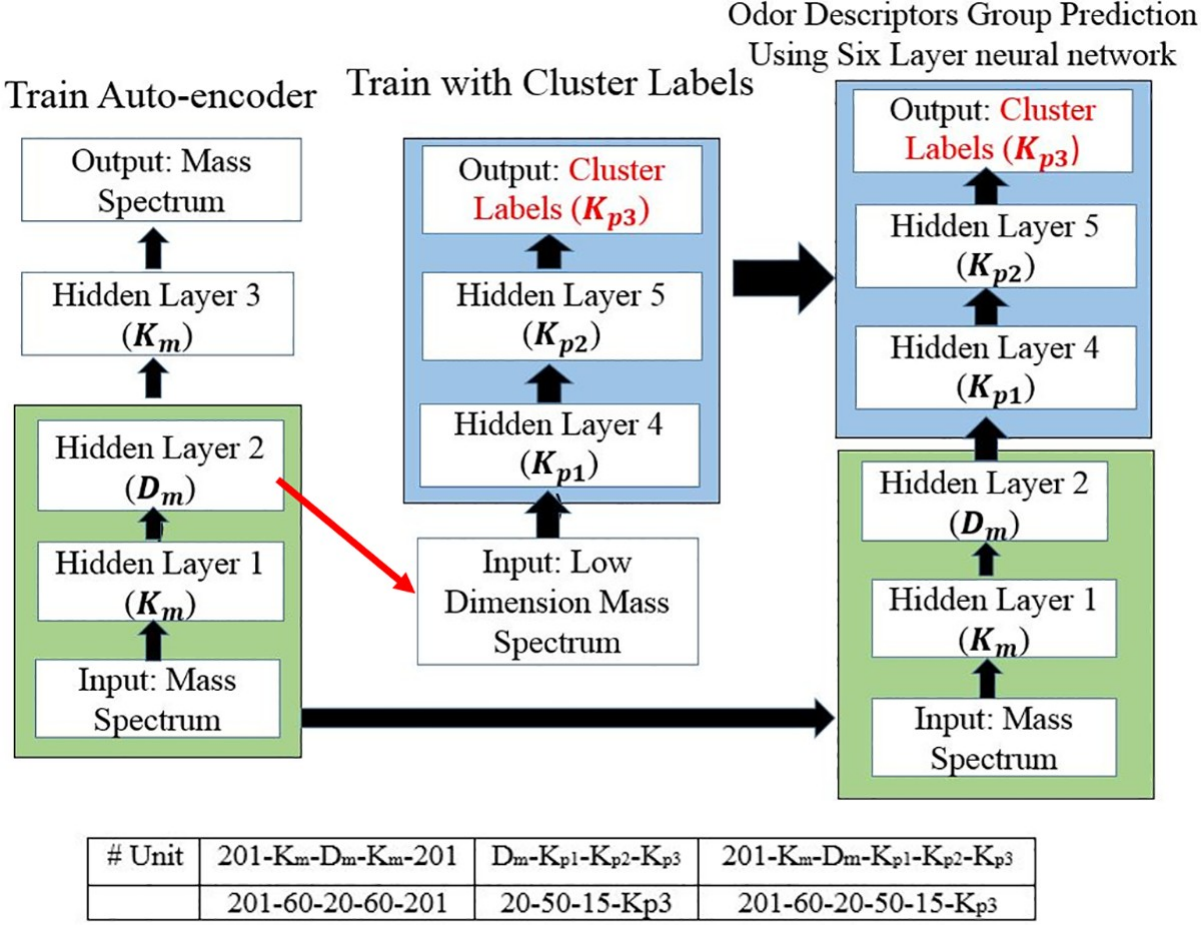
Odor descriptor group predictive model. (In Bottom shows the optimized hyper-parameter of the model).

Six layer neural network model is used in this study as shown in Fig 5 that predicts the group of odor descriptor from the mass spectrum of essential oils. We use the extracted features of mass spectrum of essential oils as input of the neural network that predicts the presence or absence of odor descriptor group as the target output. As we only want the presence or absence of odor descriptor group, the target output will be only 0 or 1. If the dimensions of the attributes are too large, then the problem of curse of dimensionality [15] can degrade the performance of the classification. As mass spectrum dataset dimension is high in this study, firstly a five layer auto-encoder was used for dimensionality reduction. The auto-encoder was trained to minimize the error for reproducing the original mass spectrum of essential oils using cross-validation (as shown in left part of Fig 5). Thus, we set 20 feature vectors from the original 201 dimensional features of mass spectrum through an optimization procedure. A six fold cross-validation procedure was used where 80 samples were used for training the model and other 16 samples were used for the evaluation of the model.

A training set of N vectors [**x**_**1**_,**x**_**2**_,…**x**_**n**_,…**x**_**N**_], each of which consists of K variables, were used as input during the optimization process. An auto-encoder with l hidden layers then calculates the output y_kn_ and updates the parameters to reduce the error function E in Eq (1), where, *λ* is the coefficient for the L2 regularization term to avoid overfitting, *β* is the coefficient for the sparsity regularization term and $w_{ji}^{\left(l\right)}$ is the weight matrix of *i* ^th^ row of j^th^ training example in the *l* ^th^ layer in the network including the biases. Encouraging sparsity of an auto-encoder is possible by adding Ω_sparsity_ to the cost function [16]. Sparsity regularizer in Eq (1) attempts to enforce a constraint on the sparsity of the output from the hidden layer.

$$E=\frac{1}{N}\sum_{n=1}^{N}\sum_{k=1}^{K}{\left(y_{kn}-x_{kn}\right)}^{2}+\lambda *\frac{1}{2}\sum_{l}\sum_{j}\sum_{i}{{(W}_{ji}^{\left(l\right)})}^{2}+\beta *\Omega_{sparsity}$$ (1)

Then, we trained the four layer neural network model by using those reduced feature vectors as input to the model to predict the existence of odor descriptor groups as shown in the middle part of (Fig 5). After the optimization procedure, these two neural network are connected to form a six layer neural network that predicts the label of the odor descriptor group from a mass spectrum (Fig 5 right). The final output of the neural network is converted to 0 or 1 by a softmax function [17]. We used sigmoid activation function in the hidden layer of both the auto-encoder and the mapping neural network. The cross entropy was used as the loss function of the mapping neural networks. Training was carried out by error backpropagation using scaled conjugate gradient [18] (trainscg in MATLAB function).

The hyper-parameters for the odor descriptor group prediction model, specifically, the number of neurons in each hidden layer, the learning rate *η*, the regularization constant *λ*, the sparsity regularization *β*, were optimized in a series of computer simulations and these results are included in Table 1. We fix the number of odor descriptor groups *K*_*p*3_ based on the experimental results.

**Table 1:** Hyper-parameters in predictive model [correlation similarity based & word similarity (fast-text) based].

Hyper-parameter	Range of Value	Optimal value
*K*_*M*_	5~80	60
*D*_*M*_	5~50	20
*K*_*p*1_	10~60	50
*K*_*p*2_	5~20	15
*K*_*p*3_	---	—
*η*	0.1~0.001	0.01
L2 regularization *λ*	0.1~0.0001	0.0001
Sparsity regularization (*β*)	1~2	1
# epochs in MS auto-encoder training		1000
# epochs in Mapping network training		1000

## Result of predictive model

**Fig 6:**
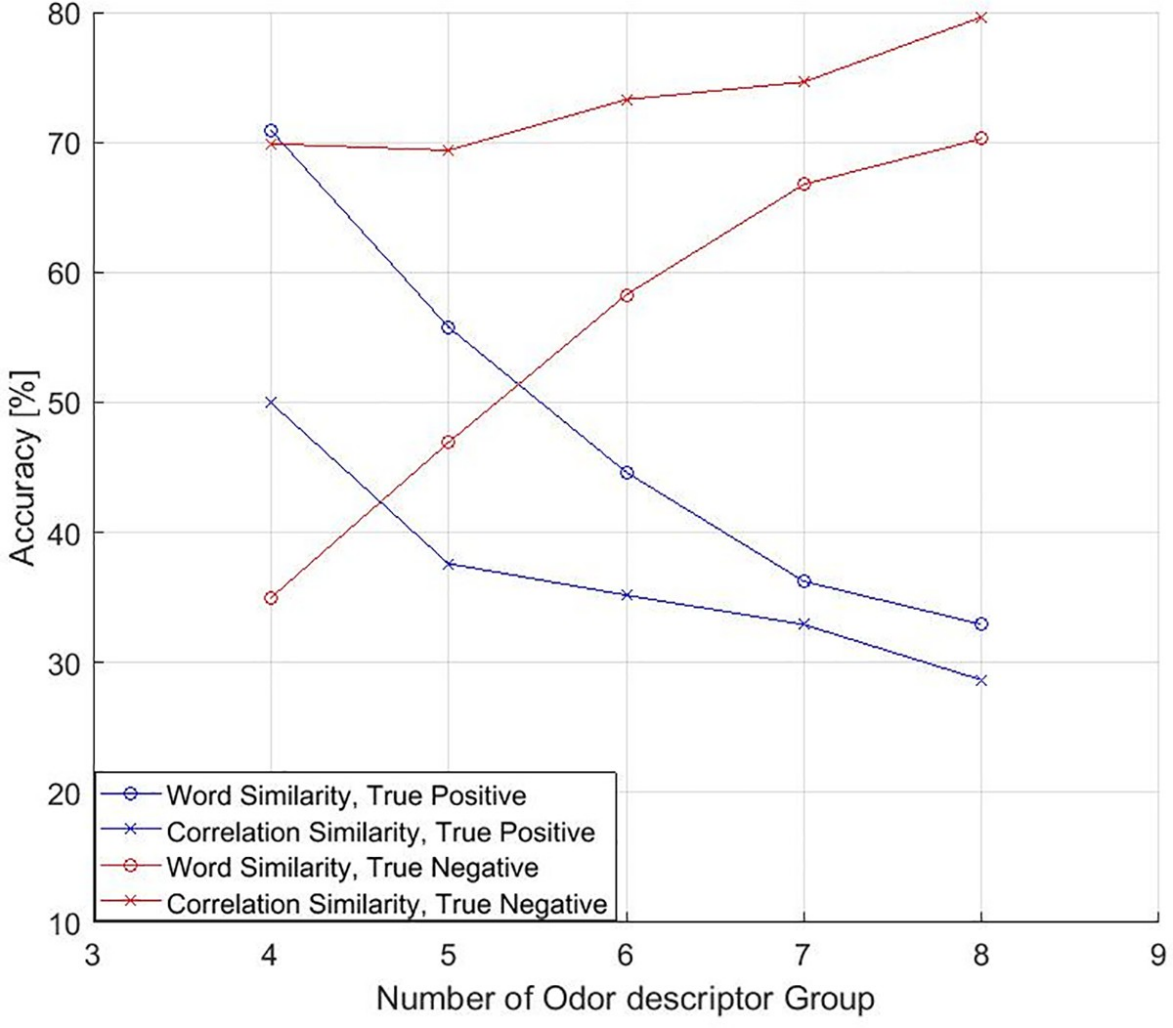
The accuracy of the predictive model of the odor descriptor group.

The number of odor descriptor groups (*K*_*p*3_) was carefully determined as the distribution of the samples influenced the viability of the model. (Fig 6) shows the accuracy of the predictive models (continuous value based correlation-coefficient and word Similarity based) as a function of number of odor descriptor group. ‘True positive’ indicates the rate at which the predicted output is 1 when the desired output is 1 and ‘true negative’ indicates the rate at which the model predicted output 0 when the desired output is 0. The predictive model tends to give the output of ‘0’ because most of the desired outputs were zeros (Imbalanced dataset with respect to positive and negative target), thus reducing the accuracy of true positives. From the results of this experiments, *K*_*p*3_ was set to 5 to ensure the classification accuracy. Table 2(A) and 2(B) show the accuracy of the predictive models based on correlation coefficient matrix expressed by continuous values and word similarity, respectively, when the number of odor descriptor group is 5.

**Table 2:** Accuracy of predictive model for odor descriptor group 5. **(a)** Continuous Value Based Correlation-coefficient; **(b)** Word Similarity (Fast-Text) [**TP** = True Positive & **TN** = True Negative].

**(a)**	TP = 178 TN = 302	**Desired Output**	
**Output**		1	0
	1	37.6%	30.62%
	0	62.4%	69.38%
**(b)**	TP = 235 TN = 245	**Desired Output**	
**Output**		1	0
	1	55.78%	53.1%
	0	44.22%	46.9%

Although the number of odor descriptor groups has a tradeoff relationship with the accuracy of the model, the prediction accuracies of true positive and true negative using clusters based on language processing (Word similarity model) were 55.78% & 46.9% respectively, when the number of odor descriptor group was 5.

Natural language semantic word similarity on a small set of odor descriptors shows better result to predict odor descriptor groups because these descriptors mapped onto the olfactory semantic domain more accurately. In Fast-Text method, plenty of data are available for evaluating the word similarity because Wikipedia corpus is used to train the Fast-Text model. On the other hand, only small number of data are available for evaluating word similarity in continuous method. Descriptors like oily, fatty, earthy, pungent, peppery and spicy made cluster more closely with each other in (Fig 3(B)) than continuous value-based correlation coefficient matrix as those descriptors are divided into several clusters. One hypothesis that describes this semantic similarity is that, semantically similar words embedded in a low dimensional vector space that are closed to each other by measuring cosine distance.

## Synthetic Minority Oversampling Technique (SMOTE)

SMOTE [19] is a statistical technique for balancing the positive and negative classes that is widely used in machine learning with imbalanced data. The new examples created by this technique are not just copies of the minority classes; instead, this algorithm takes samples of the feature space for the minor class and its nearest neighbors, and randomly generates new instances that create the aggregate properties of the target case, including its neighbors. This technique increases the features available for each class and makes the samples more general. Oversampling is used when the amount of data collected is insufficient. On the other hand, under-sampling can be used if a class of data is overrepresented majority class. Oversampling is usually preferred because under-sampling can result in significant information loss from the data.

The dataset for predicting odor descriptor group is a general unbalanced dataset because the distribution of positive samples (minority samples with specific Odor descriptor group) and negative samples (majority samples with non-specific odor descriptor group) are not uniform. This is the reason why it influenced the performance of the predictive model.

**Fig 7:**
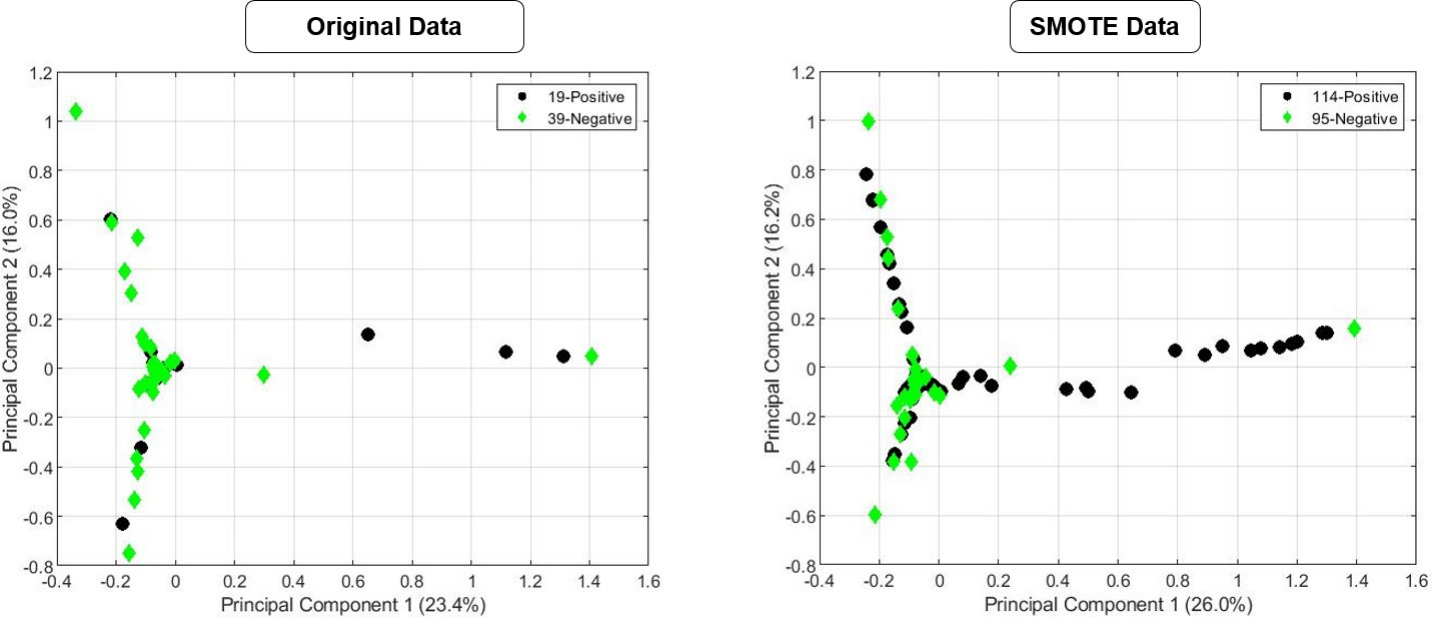
Using SMOTE to create more samples. (Here we use cluster 5 of Odor descriptor group 5 from Continuous value-based Correlation Coefficient model) (a) Original Data and (b) SMOTE data.

Approach for balancing the positive & negative classes (for both continuous value-based correlation coefficient and word similarity based models) is given below. 1) Firstly, 96 essential oil samples were divided into training set (60% for training—58 samples) and test set (38 for testing). 2) Then, SMOTE was used in 58 original samples of training data to balance the positive and negative samples. We implemented this sampling method in a function called SMOTE using Data Mining with R (DMwR) library [20]. Tables 3 and 4 shows an example of SMOTE technique over 58 training samples for each odor descriptor group of two model (for odor descriptor group 5) and used parameter for oversampling and under-sampling the original training dataset. Here, P_SMOTE and N_SMOTE indicates the number of positive and negative sampling after using SMOTE technique. A 5 fold cross validation approach was applied to training dataset (SMOTE dataset & Original Data) using previously optimized 4 layer multilayer perceptron model. Finally, the model was tested with original 38 testing dataset (in both cases testing dataset was same). Testing dataset doesn’t include the artificial data as well as the original data used for artificial data generation. We can get a better understanding of what was done by plotting the original and SMOTE’s datasets using principal component analysis (PCA) on cluster 5 of Odor descriptor group 5 from correlation Similarity based model in (Fig 7). The *SMOTE ()* function with the value 500 and 100 for the parameter oversampling and under-sampling respectively was called. This function generates synthetic similar examples to the existing minority points, thus made a larger decision boundaries that increase the generalization capabilities of classifier and improve the performance of the model.

**Table 3:** Synthetic Minority Oversampling Technique in odor descriptor group 5. (continuous value-based correlation coefficient model).

58 Original Training Dataset	Cluster 1	Cluster 2	Cluster 3	Cluster 4	Cluster 5	Total
**Positive (1)**	22	36	4	26	19	107
**negative (0)**	36	22	54	32	39	183
**over sampling**	600	500	2000	500	500	
**Under sampling**	100	120	50	120	100	
After SMOTE						Total
**P_SMOTE**	154	132	69	156	114	625
**N_SMOTE**	132	132	60	156	95	575

**Table 4:** Synthetic Minority Oversampling Technique in odor descriptor group 5. (word similarity based model).

58 Original Training Dataset	Cluster 1	Cluster 2	Cluster 3	Cluster 4	Cluster 5	Total
**Positive (1)**	21	34	50	29	11	145
**negative (0)**	37	24	8	29	47	145
**over sampling**	500	600	600	600	600	
**Under sampling**	120	120	120	120	100	
After SMOTE						Total
**P_SMOTE**	126	172	57	208	77	640
**N_SMOTE**	126	168	56	203	66	619

**Fig 8:**
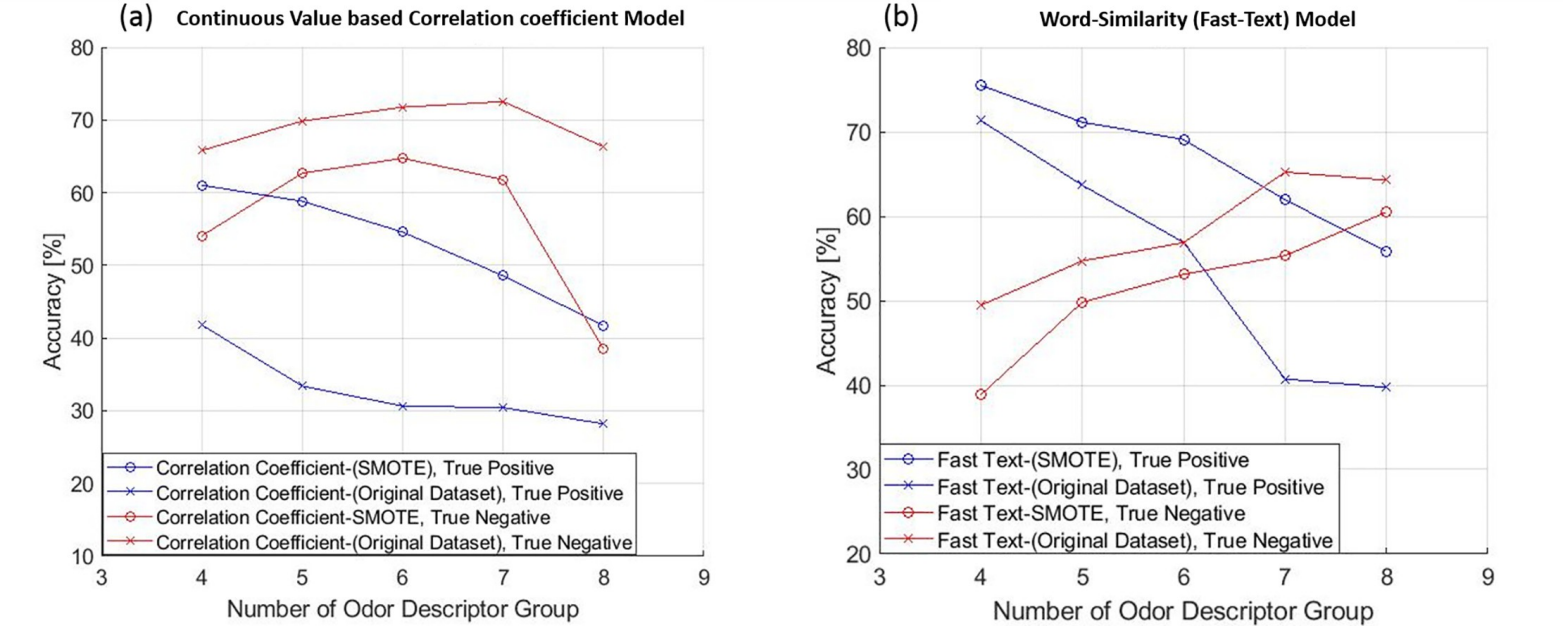
Accuracy comparison of odor descriptor group prediction using SMOTE data and original dataset for all odor descriptor group (four to eight). (a) Continuous value-based Correlation Coefficient model and (b) Word Similarity (Fast-Text) based model; where in both cases testing dataset was same.

The accuracy after SMOTE application is compared with that before SMOTE application as is shown in (Fig 8). Fig 8(A) and 8(B) show the accuracy comparison of odor descriptor group prediction using SMOTE data and original dataset for all odor descriptor group (four to eight) for correlation based and word similarity based model respectively where in both cases testing dataset was same. This experiment was performed to show that using SMOTE over an imbalanced dataset can improve the accuracy of model prediction. The prediction accuracy was improved from 33.44% to 58.82% for true positive of Continuous value-based Correlation coefficient model and 63.68% to 71.14% for true positive of word similarity based model when the number of odor descriptor group is 5.

## Conclusion

In this study, we proposed a mathematical model using machine learning with the natural language processing method (Fast-Text) to predict the human odor impression by forming odor descriptor group from mass spectra of chemical mixtures such as essential oils. Moreover, the problem of overcoming the vanishing correlation between similar odor descriptors due to the effect of binary value problem and an approach to create the similarity among odor descriptors was proposed. Although the number of odor descriptor groups has a tradeoff relationship with the accuracy of the model, the prediction accuracies of true positive and true negative using clusters based on language processing (Fast-Text model) were 55.78% & 46.9% respectively, evaluated by 6 fold cross-validation, when the number of odor descriptor group was 5, while continuous value based correlation coefficient model showed lower accuracy.

Moreover the results were improved for both models, with continuous value based correlation coefficients and word similarity, where the positive and negative classes are imbalanced. Using the SMOTE method, the prediction accuracy was improved from 33.44% to 58.82% for true positive of Continuous value-based Correlation coefficient model and 63.68% to 71.14% for true positive of word similarity based model using 5 fold cross-validation, when the number of odor descriptor group was 5. Thus, the prediction accuracy much increased when word similarity method together with SMOTE method was used.

Note that, the most difficult point to predict odor impression is small amount of the data. Especially it is very difficult to collect large scale sensory data. This point is completely different from visual and auditory fields. We achieved true positive prediction of approximately 70% even under difficult situation although there is still room for improvement. Our contribution here was to show the fundamental methods to predict odor perception using binary sensory data.

## Supporting information

S1 Table Odor descriptor table. (CSV)

S2 Table 38 odor descriptor. (CSV)

S1 Data (XLSX)
